# Familial Mediterranean Fever associated diseases in children

**DOI:** 10.1186/1546-0096-13-S1-P84

**Published:** 2015-09-28

**Authors:** ZB Özçakar, N Cakar, N Uncu, B Acar Celikel, F Yalçınkaya

**Affiliations:** 1 Ankara University, Pediatric Rheumatology, Ankara, Turkey; 2 Ankara Child Health, Hematology, Oncology Education and Research Hospital, Pediatric Rheumatology, Ankara, Turkey

## Question

Familial Mediterranean fever (FMF) is an autosomal recessive disease, characterised by recurrent, self limited attacks of fever with serositis. Certain diseases were more commonly detected in patients with FMF. The aim of our study was to investigate the frequency of FMF-associated diseases in children.

## Methods

Files of FMF patients who had been seen in two reference hospitals in Ankara, in the last two years, were retrospectively evaluated. Patients with FMF and concomitant diseases were incuded to the study.

## Results

Among 600 FMF patients 30 (18 females, 12 males; mean age 14,72 ± 5.47 years) of them (5%) were found to have a concomitant disease. Fourteen patients had juvenile idiopathic artritis; 5 had sacroilitis (3 of them had HLA B27 positivity); 6 had inflammatory bowel disease and 5 had other diseases including a patient with Behçet's disease and one with systemic lupus erythematosus. Mean age at FMF onset and associated disease onset were 54,00 ± 46,35 months and 90,46±51,65 months, respectively. 52% of the patients had homozygous M694V mutation. Classical FMF attacks were present in 26 patients; remaining 4 patients had atypical symptoms but had 2 mutations.

## Conclusions

Certain inflammatory diseases were more frequently detected in patients with FMF during childhood. In countries where FMF is prevalent clinicians dealing with FMF and other inflammatory diseases should be aware of these associations.

